# The Disease Burden of Hereditary Angioedema: Insights from a Survey in French-Canadians from Quebec

**DOI:** 10.1155/2024/3028617

**Published:** 2024-03-07

**Authors:** Jean-Nicolas Boursiquot, Hugo Chapdelaine, Charles St-Pierre, Jacques Hébert

**Affiliations:** ^1^CHU de Québec, Québec, Canada; ^2^Département de Médecine, Centre Hospitalier de l'Université de Montréal, Université de Montréal, Québec, Canada; ^3^Institut de Recherches Cliniques de Montréal, Québec, Canada

## Abstract

**Background:**

Limited data are available on the clinical profile and disease burden of hereditary angioedema (HAE) in Canadians.

**Objective:**

This study aimed to assess HAE disease characteristics and the burden of disease in Canadians with HAE types I, II, and normal levels of C1 inhibitor (nC1-INH).

**Materials and Methods:**

A 46-item patient survey evaluating clinical characteristics and burden of disease was developed and disseminated by the HAE patient organization *Angio-oédeme héréditaire du Québec* in Quebec, Canada, from May 2019 to February 2020. The survey received Research Review Board ethics approval.

**Results:**

In the 35 respondents, HAE type I was the most common (46%), followed by nC1-INH (43%). Female participants were significantly younger at first symptom presentation than males (*p*=0.04). Prior to diagnosis, 69% of participants underwent unnecessary treatments and procedures, with a 10-year delay between first symptoms and diagnosis. Before starting the current treatment, 42% of participants experienced weekly HAE attacks. Most participants identified experiencing attacks in the abdomen (89%), followed by the larynx (66%), feet (66%), hands (63%), and face (63%). Most attacks were severe or moderate, yet almost half of patients waited >1 hr before getting medical attention at their last emergency department (ED) visit. HAE was associated with decreased health-related quality of life, leading to significant functional impairment in personal and professional life. As compared to HAE type I/II, patients with HAE nC1-INH were treated more often with tranexamic acid for long-term prophylaxis, and their condition was less controlled, resulting in more attacks and ED visits.

**Conclusion:**

HAE manifests in this patient population as frequent moderate-to-severe attacks and a high disease burden; the HAE subtype may differentially affect care requirements. There is an urgent need for increased awareness and education on HAE among treating physicians.

## 1. Introduction

Hereditary angioedema (HAE) can be categorized into three types: HAE with a deficit of C1- inhibitor levels (HAE type I), HAE with dysfunctional C1-inhibitor levels (HAE type II), and HAE with normal C1-inhibitor function (HAE nC1-INH). Regardless of the type, the disease results in random and often unpredictable attacks of painful swelling typically affecting the extremities, bowel mucosa, genitals, face, and upper airway. Attacks are associated with significant functional impairment, decreased health-related quality of life (QoL), and mortality in the case of laryngeal attacks [1, 2].

Individuals with HAE may experience long delays in diagnosis and inappropriate treatment during emergency department (ED) visits [2, 3]. Even with symptoms of moderate intensity, HAE can impair daily living, productivity at work or school, and QoL [4]. The unpredictability of attacks can lead to continuous anxiety, even during symptom-free periods [5].

Data on the clinical profile and disease burden of HAE in Canada are limited in all types of HAE, particularly in patients with HAE nC1-INH. The present survey aimed to assess the disease characteristics of all three HAE types and their burden in a cohort of Canadians.

## 2. Materials and Methods

### 2.1. Questionnaire

The survey method was selected to obtain a snapshot of the clinical burden and disease characteristics at the present time. Survey questions were developed in collaboration with HAE experts to characterize the presentation of HAE and disease burden in a cohort of people in Quebec. The questionnaire used in this study was inspired by the Hereditary Angioedema Quality of Life (HAE-QoL) questionnaire proposed by Caballero et al. [5] and Prior et al. [6], which was the first disease-specific questionnaire, adopted for HAE with C1-INH deficiency. Additional questions have been incorporated to evaluate specific objectives within the study that were not fully covered by the validated questionnaire. The present 46-question survey explores demographics, presentation of HAE, resource utilization, QoL, and treatment, including on-demand versus prophylactic treatment (Appendix). The questionnaire received ethics approval from the Research Review Board Inc. (RRB ID: 2020.553).

### 2.2. Inclusion Criteria

Study participants were eligible if they were 16 years of age or older and had been diagnosed with HAE. The verification of participants' diagnoses was undertaken by the patient organization “Angioedème héréditaire du Québec” (AOHQ), which confirmed the diagnoses with the respective doctors who had initially diagnosed the individuals. It is noteworthy that all these patients are under the care of board-certified immunologists specializing in HAE.

### 2.3. Data Collection

A web-based survey was disseminated by AOHQ in Quebec, Canada, from May 2019 to February 2020. AOHQ identified 70 potential participants, 50 of whom were approached to complete the survey at the patients' annual meeting or using the AOHQ members' registry. Out of these individuals, 35 completed the survey, for a response rate of 70% (35/50). The survey was completed in person during the individual's annual meeting with AOHQ or on the phone. Informed consent was obtained from each participant before enrollment. Consent was obtained verbally if the meeting was on the phone or written if conducted in person.

### 2.4. Statistical Analysis

Statistical analysis was performed using SAS software. Descriptive statistics and frequency distributions were performed for all continuous and categorical demographics/clinical variables collected in the study. Differences in attack frequency and duration pre- and posttreatment were assessed using the Wilcoxon signed rank test (significant between-group differences at *p* ≤ 0.05). Differences in duration since the last ED visit between participants using or not using long-term prophylaxis (LTP) and differences between HAE type I/II to HAE nC1-INH were assessed using Fisher's exact test (significant between-group differences at *p* ≤ 0.05). Differences in the subgroup analysis of HAE nC1-INH with tranexamic acid for LTP were assessed using the Wilcoxon signed rank test (significant between-group differences at *p* ≤ 0.05).

## 3. Results

### 3.1. Demographics

Thirty-five participants, preponderantly female (80%), had a median age of 46 (range: 16, 73) (Table 1). At the first presentation of symptoms, female participants (median age (range) = 16 (1, 38)) were significantly younger than males (median age (range) = 46 (12, 69); *p*=0.04).

### 3.2. HAE Diagnosis and Clinical Characteristics

The majority of participants (74%) were diagnosed by an immunologist or allergist (Table 1). Most respondents (94%) reported that only some or none of their family members have been screened (Table 1). The most common HAE type was type I (46%), while 3% of participants were type II and 43% were HAE nC1-INH.

**Figure 1:**
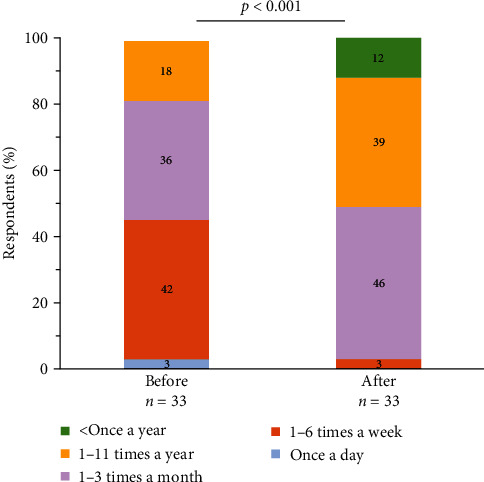
Frequency of HAE attacks before and after starting current treatment.

**Figure 2:**
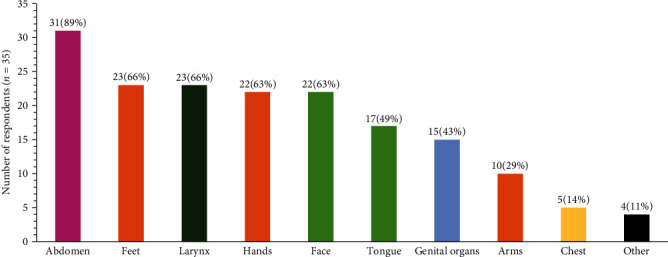
Location of HAE attacks.

**Figure 3:**
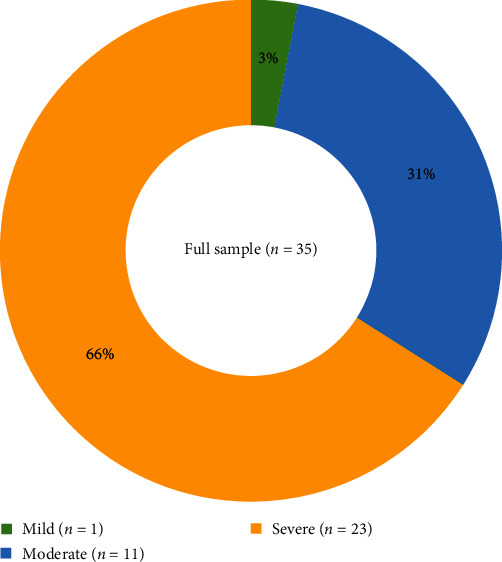
Severity of HAE attacks.

Before starting their current treatment, 42% of survey participants experienced weekly HAE attacks (defined as 1–6 attacks per week) (Figure 1). Most participants reported experiencing attacks in the abdomen (89%), followed by larynx (66%), feet (66%), hands (63%), and face (63%) (Figure 2). Most respondents categorized the severity of their attacks as severe (66%, defined as “impossible to continue current activities/requires immediate treatment”) or moderate (31%, defined as having “a perceived impact on daily activities”) (Figure 3).

### 3.3. Time to Diagnosis and Resource Utilization

Prior to diagnosis, 69% of participants reported they had undergone unnecessary treatments and procedures (Table 2), with a median delay of 10 years (range: 0, 44) between the onset of first symptoms and eventual diagnosis (Table 1). Median diagnostic delay was 7.5 years (range: 2, 44) for HAE type I/II and 11.5 years (range: 0, 29) for HAE nC1-INH respondents. The majority (70%) of participants' most recent ED visit occurred more than a year ago; about half (46%) of those surveyed waited over an hour to see a physician during that ED visit (Table 2).

### 3.4. Psychological and Professional Impact

Most participants (86%) indicated that HAE has negatively impacted their psychological and emotional well-being (Table 3). A large proportion of participants reported that HAE had negatively affected their relationship with loved ones (57%) and had forced them to give up social activities (89%).

**Figure 4:**
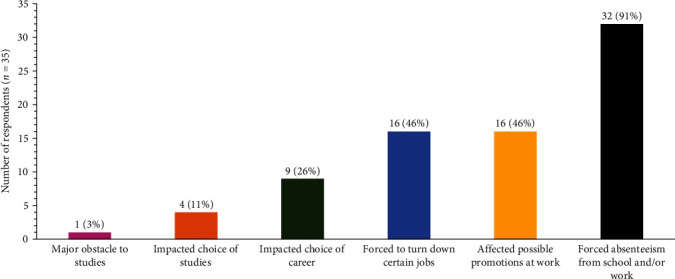
Impact of HAE on professional aspects of patient lives.

The majority of participants (80%) also reported that HAE had negatively impacted their professional life (Table 3). Of those surveyed, 91% were forced to miss school/work due to HAE (Figure 4), and 57% have been prevented from working.

### 3.5. Treatment

**Figure 5:**
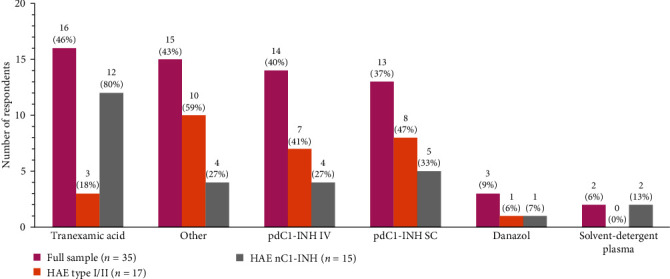
Type of treatment previously received or currently being received for treating HAE. IV, intravenous; pdC1-INH, plasma-derived C1 inhibitor; SC, subcutaneous.

Tranexamic acid (46%), subcutaneous plasma-derived C1-INH (pdC1-INH) (37%), and intravenous pdC1-INH (40%) were the most commonly reported previously or currently used treatments (on-demand or LTP) (Figure 5). Among the participants, 91% reported they self-administer the treatment at home, and 44% reported experiencing side effects while on treatment (Table 4).

Participants reported a significant decrease in attack frequency since starting their current treatment (*p*  < 0.001) (Figure 1). Before starting the current treatment, 42% of respondents experienced attacks 1–6 times a week, whereas afterward, 46% reported attacks 1–3 times a month. Additionally, 3% of survey participants identified daily attacks prior to their current treatment, but this ended once their ongoing treatment began (Figure 1).

**Figure 6:**
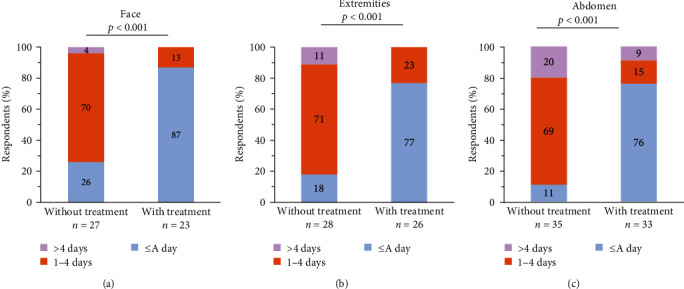
HAE attack duration in the (a) face, (b) extremities, or (c) abdomen, without and with treatment.

There was also a significant decrease in attack duration with treatment; specifically, a decrease in the duration of facial swelling (*p* < 0.001), swelling in the extremities (*p* < 0.001), and abdominal swelling with treatment compared to without treatment (*p* < 0.001) (Figure 6). Participants reported that without treatment, most attacks lasted 1–4 days in the face, extremities, and abdomen, but with treatment lasted less than a day (Figure 6).

**Figure 7:**
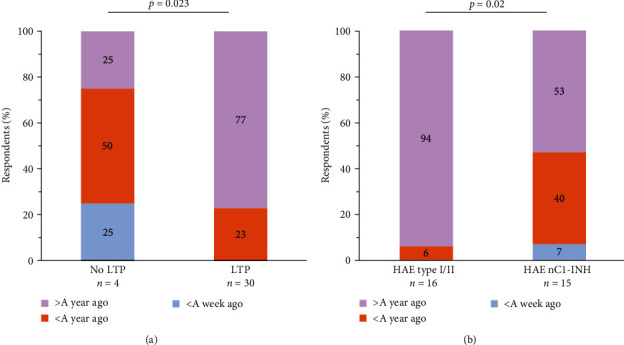
Time since the last ED visit for an HAE episode for participants (a) receiving LTP and those not receiving LTP and (b) with HAE type I/II versus HAE nC1-INH. HAE, hereditary angioedema; HAE nC1-INH, HAE with normal C1 inhibitor; LTP, long-term prophylaxis.

Most (89%) survey respondents were receiving LTP (Table 4). There was a significant difference in time since the last ED visit when comparing participants receiving LTP to those who were not (*p*=0.023) (Figure 7(a)). Most respondents receiving LTP reported that their last visit to the ED was over a year ago (77%), whereas most who did not receive LTP reported visiting the ED less than a year ago (50%).

### 3.6. Subanalysis: Comparing Outcomes in HAE Type I/II versus nC1-INH

A subanalysis was performed comparing HAE type I/II to HAE nC1-INH. No significant differences emerged in the demographics between individuals with HAE types I/II compared to HAE nC1-INH (Table 5). A higher proportion of those with HAE nC1-INH reported an ED visit within the previous year compared to individuals with type I/II (*p*=0.02) (Figure 7(b)).

For HAE nC1-INH participants, the most common previously or currently received treatment, and the most commonly received LTP, was tranexamic acid (Figure 5; Table 6). Tranexamic acid as LTP was significantly more common for HAE nC1-INH individuals than those with HAE type I/II (*p*=0.03) (Table 6). HAE type I/II participants reported that other treatments were more common as their previous or current therapy (Figure 5), with most specifying icatibant as the other type of therapy.

### 3.7. Subgroup Analysis: Comparing Outcomes in Individuals with HAE nC1-INH Using Tranexamic Acid for LTP

A subgroup analysis was performed in individuals with HAE nC1-INH who used tranexamic acid for LTP (*n* = 9). Participants reported a significant reduction in abdominal pain duration (*p*=0.03) but not in facial swelling time (*p*=0.053) or duration of swelling in the extremities (*p*=0.17) with tranexamic acid use. HAE nC1-INH participants using tranexamic acid also reported a significant reduction in attack frequency with treatment compared to before treatment (*p*=0.01). A total of 4 (50%) participants with HAE nC1-INH using tranexamic acid for LTP reported side effects. A total of 6 patients (67%) with HAE nC1-INH using tranexamic acid for LTP reported visiting the ED > a year ago; 3 (33%) reported visiting the ED < a year ago.

## 4. Discussion

This cross-sectional survey assessed the presentation of HAE and disease burden in a cohort of individuals from Quebec, Canada. This cohort includes mostly type I and HAE nC1-INH (46% and 43%, respectively), thus highlighting the experience of the latter patient subset, which, to this point, has not been well described in the literature.

Most survey participants experienced weekly attacks before receiving their current treatment; nearly all identified their average HAE attack as moderate or severe. Most respondents indicated that HAE had impaired their psychological and emotional well-being and professional lives; nearly all those surveyed reported having missed school or work due to HAE, and over half reported they have been prevented from working.

Most participants reported they were using LTP, and the use of treatment significantly decreased the duration of swelling and attack frequency. When comparing those with HAE type I/II to nC1-INH, participants with HAE nC1-INH had a longer time to diagnosis and tended to visit the ED more frequently. They also primarily used tranexamic acid for LTP. Most HAE type I/II participants used subcutaneous or intravenous C1-INH concentrate for LTP, but a minor proportion received tranexamic acid. Due to limited efficacy data, tranexamic acid is not recommended as LTP by the latest international guidelines but may be useful in certain patients when first-line LTP options are unavailable or androgen use is contraindicated [7].

The patient demographics in this study are comparable to other survey studies of people with HAE in the United States and Europe [3, 8]. The majority of survey participants were female, which aligns with the literature on HAE. There is also a difference in the age at first presentation for females compared to males, which may be due to the effect of estrogenic status on HAE. In women, the age at the first appearance of HAE symptoms often correlates with the onset of puberty; estrogen can exacerbate HAE [9].

Regarding HAE presentation, participants reported a high prevalence of abdominal attacks; this is consistent with previous studies that reported frequencies ranging from 41% to 97% [2, 10, 11]. Moreover, about two-thirds of respondents experienced laryngeal attacks, which is within a similar range to what is reported in the HAE literature [11, 12]. Finally, the median diagnostic delay for survey participants was 10 years, which is also comparable to other studies in Europe and the United States reporting delays of about 9–15 years [3, 10]. Nonetheless, this delay in diagnosis underlines the need for physician education on HAE in the ED and elsewhere.

Previous studies report HAE type I as the more common form of HAE [2]; however, the prevalence of HAE nC1-INH may be underestimated due to the relative novelty of this diagnosis, as well as the lack of confirmatory tests or (in most cases) defined genetic markers for this potentially heterogeneous disorder [13, 14]. Six genetic mutations are associated with HAE nC1-INH, but there are also many instances where the genetic cause is unidentified [7]. Thus, a highlight of this study is the high prevalence of HAE nC1-INH participants. A recent HAE patient registry from the Canary Islands, Spain, also reported a higher frequency of HAE nC1-INH (34%) [15], which illustrates the need for more studies aimed at better defining the HAE nC1-INH population.

A limitation of this study is the reliance on patient self-report, as the survey results are patient-reported with no mechanism for verifying the claims in the physicians' records. The survey format is also prone to recall bias, which can lead to under- or overestimations of outcomes. An additional limitation is the lack of details captured regarding treatment side effects—severity or specific side effects were not evaluated—but based on the literature, side effects can be assumed to have been generally mild [16]. Similarly, the degree to which HAE negatively impacted QoL could not be specified, as participants experiencing minor or major impacts on different aspects of their QoL would respond in the same manner by selecting an affirmative response.

The results of this survey suggest that using LTP reduces time since the last ED visit, as well as attack frequency and duration but not severity. Since respondents identified negative impacts on QoL, one interpretation is that ongoing attacks were still perceived as burdensome despite being less frequent and shorter in duration. Since the survey was conducted between May 2019 and February 2020, this could, in part, be explained by respondents having limited or no access to other LTPs that are now available in Canada, such as lanadelumab (approved September 2018) [17] or berotralstat (approved June 2022) [18]. Importantly, lanadelumab and berotralstat are also recommended by guidelines as first-line LTP; thus, the high burden experienced by respondents may have indicated a need to consider switching LTP to help optimize treatment and improve efficacy [7].

This study provides a snapshot of the impact of HAE on the daily lives of Canadian patients. To our knowledge, no prior studies have compared the burden of type I/II HAE to HAE nC1-INH. However, the use of tranexamic acid in the present study by participants with nC1-INH HAE is in keeping with previous reports [13]. The availability of more effective treatments may lead to a substantial lessening of disease burden; thus, future research is needed to assess the impact of new treatments on disease severity and QoL. Better characterization of patient subgroups with nC1-INH HAE will improve the identification and understanding of the pathophysiology behind each group in order to find more tailored and effective therapies.

In conclusion, HAE presentation in Canadians is characterized by frequent moderate to severe attacks and a high burden of disease. Individuals face barriers at each step, from diagnosis to treatment and disease management. This indicates an urgent need for increased awareness and education on HAE among Canadian physicians, as well as continued advancements in treatment. The questionnaire from this study can be used to identify opportunities for improvements in HAE patient care. HAE type may differentially affect care requirements; additional exploration into HAE nC1-INH characterization and treatment options is an important next step for advancing patient outcomes.

## Figures and Tables

**Table 1:** Demographics and clinical characteristics.

Characteristic	Full sample (*n* = 35), median (min, max) or *n* (%)
Age (years)	46 (16, 73)
Sex	
Male	7 (20%)
Female	28 (80%)
Marital status	
Common-law	9 (26%)
Married	16 (46%)
Single	10 (29%)
Employed	19 (54%)
HAE type	
Type I	16 (46%)
Type II	1 (3%)
HAE nC1-INH	15 (43%)
Unknown	3 (9%)
Age at first HAE symptoms (years)^†^	16 (1, 69)
Age at HAE diagnosis (years)	34 (2, 69)
Diagnostic delay (years)^‡^	10 (0, 44)
Diagnosing doctor	
Internist	6 (17%)
Immunologist/allergist	26 (74%)
Gastroenterologist	1 (3%)
Hematologist/oncologist	1 (3%)
Dermatologist	1 (3%)
Family screening	
All	1 (3%)
Only some members	20 (57%)
No	13 (37%)
Do not know/NA	1 (3%)
Family deaths due to HAE	4 (11%)

HAE, hereditary angioedema; HAE nC1-INH, HAE with normal C1 inhibitor; NA, not applicable. ^†^One participant did not respond to this question (*n* = 34). ^‡^Two participants were excluded from the calculation as one respondent with HAE nC1-INH did not report their age at first symptom, and another participant with HAE type I identified as having received their diagnosis prior to experiencing their first symptom (*n* = 33).

**Table 2:** Survey questions on unnecessary treatments prior to diagnosis and resource utilization.

Question	Full sample (*n* = 35), *n* (%)
Unnecessary treatment/procedure before diagnosis	
Yes	24 (69%)
No	11 (31%)
Time since the last ED visit^†^	
<A week	1 (3%)
<A year	9 (26%)
>A year	24 (71%)
Time spent in ED before meeting doctor	
10–30 min	8 (23%)
30–60 min	11 (31%)
>60 min	16 (46%)

ED, emergency department. ^†^One participant did not respond to this question (*n* = 34).

**Table 3:** Impact of HAE on different quality of life aspects.

Does HAE negatively impact	Full sample (*n* = 35)	
	Yes, *n* (%)	No, *n* (%)
Psychological/emotional health	30 (86%)	5 (14%)
Daily activities	31 (89%)	4 (11%)
Professional life	28 (80%)	7 (20%)

HAE, hereditary angioedema.

**Table 4:** Treatment-related survey questions.

Question	Full sample (*n* = 35), *n* (%)
Method of administration	
Self-administer	32 (91%)
Family/friend administers	1 (3%)
Emergency room	2 (6%)
Side effects^†^	
Yes or sometimes	15 (44%)
No	19 (56%)
Use of LTP	
Yes	31 (89%)
No	4 (11%)

LTP, long-term prophylaxis. ^†^One participant did not respond to this question (*n* = 34).

**Table 5:** Demographic data of HAE type I/II and HAE nC1-INH.

Characteristic	HAE type I/II (*n* = 17), median (min, max) or *n* (%)	HAE nC1-INH (*n* = 15), median (min, max) or *n* (%)	*p* Value^†^
Age (years)	57 (16, 73)	43 (17, 72)	0.17
Sex	—	—	0.09
Male	6 (35%)	1 (7%)	—
Female	11 (65%)	14 (93%)	—
Marital status	—	—	0.90
Common-law	5 (29%)	3 (20%)	—
Married	8 (47%)	7 (47%)	—
Single	4 (24%)	5 (33%)	—
Employed	10 (59%)	6 (40%)	0.48

HAE, hereditary angioedema; HAE nC1-INH, hereditary angioedema with normal C1 inhibitor. ^†^*p* ≤ 0.05 is deemed significant.

**Table 6:** Types of LTP treatment used in HAE type I/II compared to HAE nC1-INH.

	HAE type I/II (*n* = 17), *n* (%)	HAE nC1-INH (*n* = 15), *n* (%)
Treatment(s) you have received as LTP		
Tranexamic acid	3 (18%)	9 (60%)
SC C1-INH concentrate	9 (53%)	3 (20%)
IV C1-INH concentrate	7 (41%)	3 (20%)
Other	3 (18%)	0 (0%)

C1-INH, C1 inhibitor; HAE, hereditary angioedema; HAE nC1-INH, hereditary angioedema with normal C1 inhibitor; IV, intravenous; LTP, long-term prophylaxis; SC, subcutaneous.

## Data Availability

Data will be available upon reasonable request.

## Supplementary Materials

Supplementary Material The supplementary file accompanying the manuscript consists of the AOH Quality of Life Questionnaire designed for HAE patients in Quebec, which served as the survey instrument for the study participants.
