# Supplementary material for: The Disease Burden of Hereditary Angioedema: Insights from a Survey in French-Canadians from Quebec
Source: J Immunol Res. 2024 Mar 7;2024:3028617. doi: 10.1155/2024/3028617 (PMC10940028; doi:10.1155/2024/3028617)
Supplement: Supplementary Material — The supplementary file accompanying the manuscript consists of the AOH Quality of Life Questionnaire designed for HAE patients in Quebec, which served as the survey instrument for the study participants. [file 3028617.f1.docx]

**Appendix**

**AOH Quality of Life Questionnaire for HAE patients in Quebec**

**Demographic data**

1. What is your gender?
   1. Male
   2. Female
2. How old are you?
   1. Years _____
3. Marital status
   1. Single
   2. Common law partner
   3. Married
4. Are you employed?
   1. Yes
   2. No
5. Does your illness prevent you from working?
   1. Yes
   2. No
6. Where do you currently live?
   1. Bas-Saint-Laurent [Lower St. Lawrence]
   2. Saguenay-Lac-Saint-Jean
   3. Capitale nationale [Québec City region]
   4. Mauricie
   5. Estrie
   6. Montreal
   7. Outaouais
   8. Abitibi-Témiscamingue
   9. Côte-Nord [North Shore]
   10. Nord-du-Québec
   11. Gaspésie-Îles-de-la-Madeleine [Gaspésie‒Magdalen Islands]
   12. Chaudière-Appalaches
   13. Laval
   14. Lanaudière
   15. Laurentides [Laurentians]
   16. Montérégie
   17. Centre-du-Québec
   18. Other
7. Which region are you from?
   1. Bas-Saint-Laurent [Lower St. Lawrence]
   2. Saguenay-Lac-Saint-Jean
   3. Capitale nationale [Québec City region]
   4. Mauricie
   5. Estrie
   6. Montreal
   7. Outaouais
   8. Abitibi-Témiscamingue
   9. Côte-Nord [North Shore]
   10. Nord-du-Québec
   11. Gaspésie-Îles-de-la-Madeleine [Gaspésie‒Magdalen Islands]
   12. Chaudière-Appalaches
   13. Laval
   14. Lanaudière
   15. Laurentides [Laurentians]
   16. Montérégie
   17. Centre-du-Québec
   18. Other

**Diagnosis**

1. At what age did you first show signs of angioedema?
   1. ___years
2. At what age did you get diagnosed with HAE?
   1. ___years
3. Have any other family members (your parents, siblings, children) been screened for the disease?
   1. All
   2. Only some members
   3. No
   4. I don’t know/does not apply
4. Has a family member (your parents, siblings, children) died as a result of an HAE episode?
   1. Yes
   2. No
   3. I don’t know/does not apply
5. What is the area of specialization of the doctor who diagnosed HAE?
   1. Family doctor
   2. Internist
   3. Hematologist-oncologist
   4. Immunology/allergy specialist
   5. ENT specialist
   6. Dermatologist
   7. Nephrologist
   8. Other (specify:____)
6. How many times in the past year have you met with the doctor primarily responsible for managing your HAE?
   1. Never
   2. Once
   3. 2–5 times
   4. 6–10 times
   5. More than 10 times
7. What type of angioedema do you have?
   1. HAE type 1
   2. HAE type 2
   3. HAE type 3/HAE with normal C1-INH
   4. Acquired angioedema
   5. I don’t know
8. Do you think that the healthcare professionals involved in your care used unnecessary treatments and procedures (e.g., surgery) before the diagnosis of HAE was confirmed?
   1. Yes
   2. No
9. Identify the location of your HAE attacks so far (select all that apply)
   1. Abdomen (stomach pain)
   2. Chest
   3. Genital organ
   4. Hands
   5. Arms
   6. Feet
   7. Face
   8. Tongue
   9. Larynx (throat)
   10. Other (specify:___)
10. Which of the following do you consider to be precipitating factors in your HAE attacks? (select all that apply)
    1. Hormone derived medicinal products (anovulants, hormone replacement therapy)
    2. Anti-hypertensive medication
    3. Anti-inflammatory medication
    4. Other medications
    5. Physical trauma (accident, dental examination, etc.)
    6. Menstrual cycle
    7. Stress
    8. Infections
11. Without treatment, what is the average duration of your facial swelling episodes (including the tongue and lips)?
    1. Less than an hour
    2. Between 1 and 12 hours
    3. Between 12 and 24 hours
    4. Between 1 and 2 days
    5. Between 2 to 4 days
    6. More than 4 days
    7. Does not apply
12. Without treatment, what is the average duration of your swelling episodes in the extremities (hands, feet, other)?
    1. Less than an hour
    2. Between 1 and 12 hours
    3. Between 12 and 24 hours
    4. Between 1 and 2 days
    5. Between 2 to 4 days
    6. More than 4 days
    7. Does not apply
13. Without treatment, what is the average duration of your stomach pain episodes (abdominal episode)?
    1. Less than an hour
    2. Between 1 and 12 hours
    3. Between 12 and 24 hours
    4. Between 1 and 2 days
    5. Between 2 to 4 days
    6. More than 4 days
    7. Does not apply
14. With treatment, what is the average duration of your facial swelling episodes (including the tongue and lips)?
    1. Less than an hour
    2. Between 1 and 12 hours
    3. Between 12 and 24 hours
    4. Between 1 and 2 days
    5. Between 2 to 4 days
    6. More than 4 days
    7. Does not apply

1. With treatment, what is the average duration of your swelling episodes in the extremities (hands, feet, other)?
   1. Less than an hour
   2. Between 1 and 12 hours
   3. Between 12 and 24 hours
   4. Between 1 and 2 days
   5. Between 2 to 4 days
   6. More than 4 days
   7. Does not apply
2. With treatment, what is the average duration of your stomach pain episodes (abdominal episode)?
   1. Less than an hour
   2. Between 1 and 12 hours
   3. Between 12 and 24 hours
   4. Between 1 and 2 days
   5. Between 2 to 4 days
   6. More than 4 days
   7. Does not apply
3. In terms of severity, you would say your average HAE attacks are:
   1. Mild (few symptoms and little impact on your daily activities)
   2. Moderate (perceived impact on your daily activities)
   3. Severe (impossible to go about your activities, requires immediate treatment)

1. Does HAE have a negative psychological and emotional impact on your life?
   1. Yes
   2. No
2. Does HAE have a negative impact on your daily activities (school, work, holidays, family life)?
   1. Yes
   2. No
3. Does HAE have a negative impact on your life professionally (work and / or studies)?
   1. Yes
   2. No
4. Are you worried about having children, or that your children might inherit your disease?
   1. Yes
   2. No
5. Do you have any side effects related to the treatment of your HAE (e.g., pain at the injection site, nausea, fatigue, etc.)?
   1. Yes
   2. No
6. Regarding your HAE, which of the following statements do you find most disturbing (many possible answers)?
   1. Possibility of a severe or even fatal attack
   2. Side effects from HAE medication
   3. Impossibility of curing the disease
   4. Risk of transmitting this disease to his children
   5. Rare disease not well known to doctors
   6. Unpredictability of the occurrence of attacks
7. Regarding the professional impact of HAE in your life, this disease (many possible answers):
   1. Had an impact on the choice of your studies
   2. Was a major obstacle to studies
   3. Has prevented you from completing your academic studies
   4. Has had an impact on your choice of career
   5. Has forced you to turn down certain jobs
   6. Has affected you in terms of possible promotions at work
   7. Has forced you to be absent from school and/or work
8. Regarding the psychological impact of HAE on your life, this disease (select all that apply):
   1. Has affected your relationship with your loved ones (family and friends)
   2. Has forced you to give up some social activities
   3. Has a slight impact on your morale
   4. Has a major impact on your morale

**Treatment**

1. Are you receiving prophylaxis (a given treatment for PREVENTING attacks) on a regular basis?
   1. Yes
   2. No
2. If you answered YES to the previous question, what treatment (several possible answers)?
   1. Danazol (Cyclomen)
   2. Tranexamic acid (Cyklokapron)
   3. C1 inhibitor concentrate (Berinert, Haegarda, Cinryze) subcutaneous
   4. C1 inhibitor concentrate (Berinert, Cinryze) Intravenous
   5. Other (specify: )
3. Which treatment do you use to treat your HAE episodes by self-administration (many possible answers)?
   1. Danazol (Cyclomen)
   2. Tranexamic acid (Cyklokapron)
   3. C1 inhibitor concentrate (Berinert, Haegarda, Cinryze) subcutaneous
   4. C1 inhibitor concentrate (Berinert, Cinryze) Intravenous
   5. Other (specify:___)
   6. I do not have any treatment that I can self-administer
4. How frequent were your HAE attacks before starting on your current treatment?
   1. Once a day
   2. 2–6 times a week
   3. Once a week
   4. 2–3 times a month
   5. Once a month
   6. 2–11 times a year
   7. Once a year
   8. Less than once a year
5. How often have you had attacks since your current treatment began?
   1. Once a day
   2. 2–6 times a week
   3. Once a week
   4. 2–3 times a month
   5. Once a month
   6. 2–11 times a year
   7. Once a year
   8. Less than once a year
6. From the following list, indicate which treatment(s) you have received or are currently receiving specifically for the treatment of your HAE (many possible answers).
   1. Tranexamic acid (Cyklokapron)
   2. Ecallantide (Kalbitor)
   3. Solvent-detergent plasma
   4. C1 inhibitor concentrate (Berinert, Haegarda, Cinryze) subcutaneous
   5. C1 inhibitor concentrate (Berinert, Cinryze) Intravenous
   6. Corticosteroids (prednisone)
   7. Other (specify:___)
   8. I do not know / do not apply

1. How many times in the past year have you consulted the emergency room in connection with your HAE?
   1. None
   2. Once
   3. 2–5 times
   4. 6–10 times
   5. More than 10 times
2. When was the last time you visited the emergency room for an HAE episode?
   1. Less than a week ago
   2. Less than a month ag
   3. Less than one year ago
   4. More than one year ago
3. During your last emergency room visit for an HAE episode, how long did it take before you met with a doctor?
   1. Less than 10 minutes
   2. Between 10 and 30 minutes
   3. Between 30 minutes and 1 hour
   4. More than 1 hour
4. Do you believe you were adequately treated during your visit to the emergency room?
   1. Yes
   2. No

1. During an HAE episode requiring treatment, which statement best describes your situation (only one possible answer):
   1. I self-administer treatment at home
   2. A member of my family administers my treatment
   3. I go to CLSC (local community service centre) or a nurse visits me at home to administer my treatment
   4. I go to the emergency room
2. In case of an attack, are you able to self-administer the treatment?
   1. Yes
   2. No
3. What would you consider to be the ideal treatment to prevent HAE attacks (1 possible answer)?
   1. Medicine administered orally every day
   2. Medicine injected subcutaneously every 2 weeks
   3. Medication injected intravenously every 4 weeks
4. Do you believe that one day there will be a cure for HAE?
   1. Yes
